# Low serum paraoxonase1 activity levels predict coronary artery disease severity

**DOI:** 10.18632/oncotarget.14305

**Published:** 2016-12-27

**Authors:** Ting Sun, Jingchao Hu, Zhaofang Yin, Zuojun Xu, Liang Zhang, Li Fan, Yang Zhuo, Changqian Wang

**Affiliations:** ^1^ Department of Cardiology, Shanghai Ninth People's Hospital, Shanghai JiaoTong University School of Medicine, Shanghai 200025, China

**Keywords:** paraoxonase1, coronary artery disease, atherosclerosis, high-density lipoprotein cholesterol

## Abstract

Paraoxonase1 (PON1) activity is closely related to coronary artery disease (CAD). However, whether PON1 activity can predict the degree of coronary stenosis remains unknown. In the present study, the serum PON1 activity and related factors that influence PON1 activity were analyzed in 186 patients with diagnostic coronary angiography. The serum PON1 activity was determined using a spectrophotometry-based assay in 186 patients with diagnostic coronary angiography, in which coronary stenosis severity was graded and clinically defined as single- or multi-vessel stenosis >50%. Target lesion stenosis was quantified via quantitative coronary angiography (QCA). The serum PON1 activity was significantly decreased in the CAD group, the multiple coronary stenosis subgroup, and the diabetes mellitus subgroup compared with each control group. The PON1 activity was positively correlated with the High density lipoprotein cholesterol (HDL-C) and Apolipoprotein A1 (ApoA1). Males, smoking, diabetes, and heart failure were identified as factors that influenced PON1 activity. Furthermore, a Receiver Operating Characteristic Curve (ROC) analysis indicated that a PON1 activity cut-off point of 330 U/L could predict CAD with a sensitivity of 52% and a specificity of 65%. In conclusion, low PON1 activity predicted the degree of coronary lesion, particularly in multiple vessel lesions, smokers, and diabetes, which may represent a biochemical marker for the severity of CAD.

## INTRODUCTION

Coronary artery disease (CAD) is increasing and has become the leading cause of death in China. There are many risk factors for CAD, including hypertension, diabetes mellitus, hyperlipidemia, stress, depression, family history, smoking and alcoholism [1–3]. In contrast to these risk factors, High-density lipoprotein-cholesterol (HDL-C) is strongly and inversely correlated with atherosclerotic cardiovascular disease. Initial attention focused on the role of HDL-C in reverse-cholesterol transport. However, an increasingly greater number of studies have suggested more diverse mechanisms [4–6]. The cardioprotective effects of HDL-C result from antioxidant, anti-inflammatory and antithrombotic properties and promote endothelial repair [4–6].

The antioxidant activity of HDL-C is primarily a result of paraoxonase 1 (PON1), which is located in the serum and binds to HDL-C in a calcium dependent manner [7]. PON1 retards or reverses atherosclerosis via the prevention of low-density lipoprotein cholesterol (LDL-C) oxidation or metabolism of oxidized LDL-C (ox-LDL); it is therefore considered a major factor in the antioxidative activity of HDL-C [8–10].

In the previous decade, several studies have investigated the associations between PON1 activity and CAD susceptibility. Shekhanawar M et al. identified the serum PON1 activity levels in 50 controls and 60 CAD cases and compared the PON1 activity with total cholesterol and triglycerides [11]. The authors determined that the serum PON1 activity levels (p<0.001) were significantly lower in the CAD cases compared with the controls, and there was a negative correlation between PON1 activity and total cholesterol and triglycerides. Dullaart RP et al. measured PON1 activity, plasma apolipoprotein E (apoE) and serum amyloid A in subjects without or with metabolic syndrome and determined higher apoE levels may confer higher PON-1 activity [12]. Sagit M et al. also indicated that low PON1 is an independent risk factor for new coronary events, independent of all other risk factors, including HDL-C [13]. These studies have all demonstrated significant associations between lower PON1 activity and CAD.

However, it remains unknown whether this low PON1 activity is correlated with CAD severity. To investigate the relationship between PON1 activity and CAD, nested case control studies of patients with CAD were performed to establish a comprehensive picture of PON1 activity in CAD and to evaluate whether serum PON1 activity may be used as a biochemical marker for CAD severity.

## RESULTS

### Subject characteristics

Of the 186 participants (105 males, 81 females, mean age 67.4 ± 11.6 years, minimum 45, maximum 80), there were 63 patients with coronary arteries stenosis below 50% and 123 patients with stenosis above 50% based on the diagnostic angiography results. The demographic, clinical and baseline biochemical characteristics of the study populations are shown in Table 1. The CAD group had more patients with diabetes mellitus (DM) or hypertension, whereas the non-CAD group had more female and young patients. Smokers and alcohol consumers were similar between the study groups. There was no significant difference between the frequencies of heart failure between the groups. The CAD patients had significantly lower cholesterol, LDL-C, HDL-C, and apolipoprotein A1 levels compared with the non-CAD patients. Serum triglycerides, apolipoprotein B, and apolipoprotein A1/B were not significantly different between the groups.

**Table 1:** Demographic and clinical characteristics of the study sample according to coronary arteriography

Variable	Total (n=186)	The results of coronary arteriongraphy		*P* Value
		Non-CAD (n=63) (Stenosis <50%)	CAD (n=123) (Stenosis ≥50%)	
Age, y	67.4±11.6	62.7±10.7	69.7±11.3	0.000
Gender (male), %	56.5 (105/186)	44.4 (28/63)	62.6 (77/123)	0.018
Smokers, %	28.5 (53/186)	19.0 (12/63)	33.3 (41/123)	0.410
Alcohol consumers,%	15.6 (29/186)	17.5 (11/63)	14.6 (18/123)	0.615
Diabetes mellitus, %	27.4 (51/186)	15.9 (10/63)	33.3 (41/123)	0.015
Hypertension, %	62.9 (117/186)	47.6 (30/63)	70.7 (87/123)	0.002
NYHA class III or IV, %	33.3 (62/186)	28.6 (18/63)	35.8 (44/123)	0.324
Lipid drugs medication, %	83.3 (149/186)	71.4 (45/63)	89.4 (110/123)	0.002
Blood glucose, mmol/L	5.51±1.71	5.28±1.48	5.62±1.82	0.065
Serum creatinine, umol/L	93.0±25.4	82.0±18.3	98.6±26.8	0.000
Blood urea nitrogen, mmol/L	6.02±2.25	5.69±2.21	6.19±2.25	0.121
cardiac troponin I, ng/L	1.60±8.50	0.17±0.83	2.35±10.43	0.000
Myoglobin, μg/L	90.4±369	25.6±13.6	123.6±450.7	0.000
CK-MB, IU/L	28.5±61.7	16.1±6.0	34.9±75.0	0.282
Total cholesterol, mmol/L	4.23±1.06	4.50±0.99	4.08±1.07	0.008
LDL cholesterol, mmol/L	2.64±0.89	2.82±0.77	2.54±0.94	0.019
HDL cholesterol, mmol/L	1.09±0.3	1.14±0.27	1.06±0.32	0.023
Serum triglycerides, mmol/L	1.61±1.03	1.69±1.27	1.56±0.88	0.903
Apolipoprotein A1, g/L	1.08±0.16	1.11±0.16	1.06±0.20	0.033
Apolipoprotein B, g/L	0.86±0.22	0.87±0.22	0.85±0.23	0.400
Apolipoprotein A1/B	1.38±0.40	1.39±0.36	1.37±0.42	0.599
Paraoxonase1 activity, U/L	351±123	380±133	336±115	0.026

Values are expressed as mean ± standard deviation or percentage

### PON1 activity and HDL-C cholesterol level

**Figure 1:**
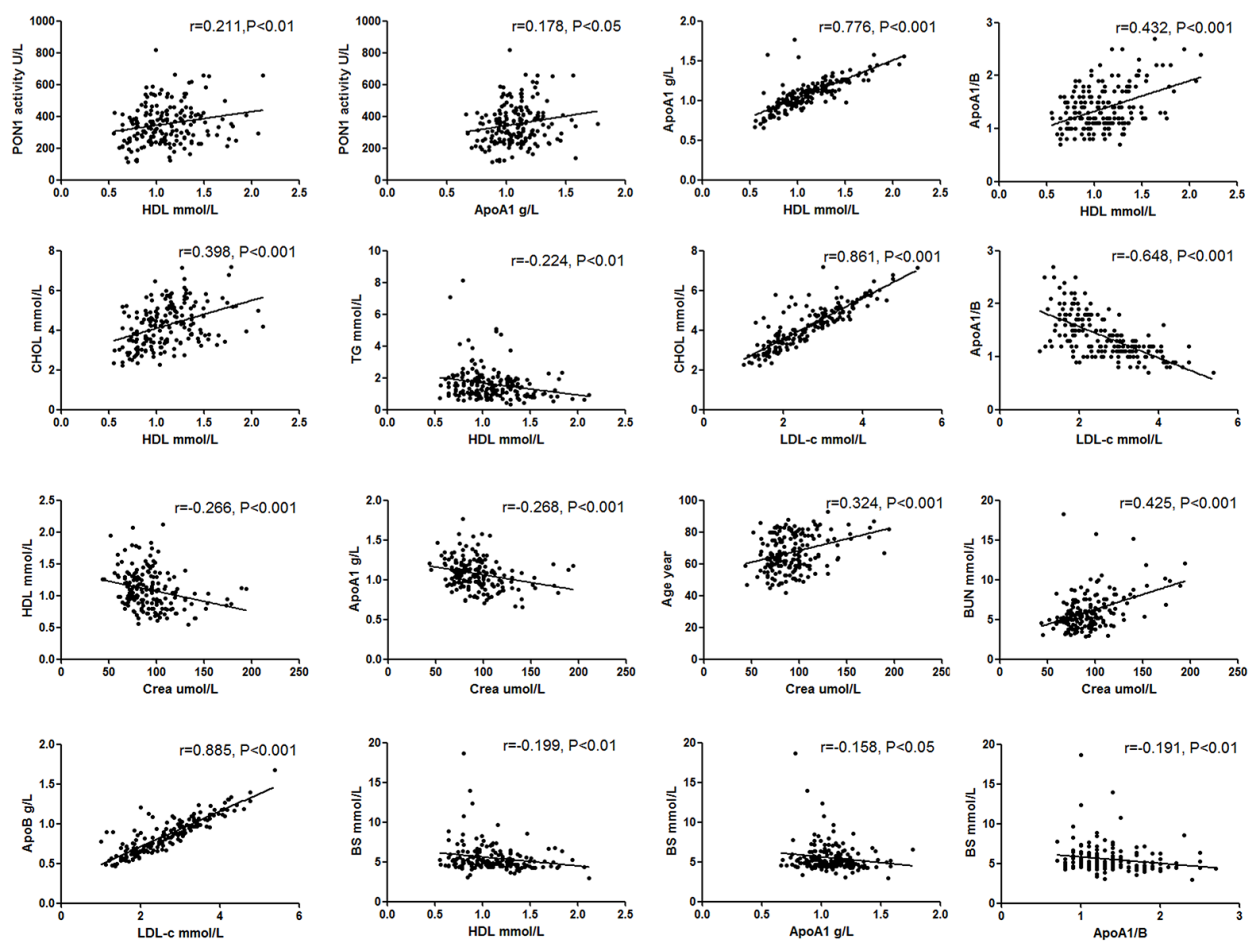
Association between serum PON1 activity and clinical characteristics and lipid parameters

The correlations among serum PON1 activity and blood lipid parameters are presented in Figure 1. A significant correlation was identified between the PON1 activity and HDL-C level. The patients with low HDL-C levels exhibited a more substantial reduction in PON1 activity, as indicated by the Pearson's correlation analysis. There was no significant difference between PON1 activity and other lipid parameters (LDL-C, Total cholesterol, Triglycerides, or Apo B). However, HDL-C was positively correlated with ApoA1, ApoA1/B, and Total cholesterol and negatively correlated with creatinine and blood glucose.

### PON1 activity and CAD severity

The serum PON1 activity was lower in the CAD group (336 U/L) compared with the non-CAD group (380 U/L) (Table 1). The PON1 activity and blood lipid levels in the different stenosis groups are presented in Table 2. The PON1 activity significantly decreased as the coronary vascular stenosis degree increased among multiple groups (P<0.05). Compared with any two groups, the PON1 activity was significantly different between the stenosis <10% and stenosis 10-50% groups (P<0.05); however, there was no difference between the stenosis 10-50% and stenosis 50-75% groups or between the stenosis 50-75% and stenosis >75% groups. The lipid levels were not significantly different among the different degrees of coronary vascular stenosis.

**Table 2:** PON1 activity and lipid levels of the study sample according to coronary vascular stenosis degree

Variable	The Degree of Vascular Stenosis						
	<10% (n=30)	10~50% (n=33)	50~75% (n=33)	>75% (n=90)	F	*P*	*P_1_*
PON1 activity, U/L	419±127	344±130	345±128	333±111	10.55	0.014	0.022
Total cholesterol, mmol/L	4.45±1.04	4.56±0.95	4.24±1.05	4.02±1.08	8.27	0.041	0.536
LDL cholesterol, mmol/L	2.69±0.72	2.92±0.81	2.61±0.85	2.52±0.97	6.79	0.079	0.223
HDL cholesterol, mmol/L	1.19±0.24	1.10±0.29	1.06±0.30	1.06±0.32	7.17	0.067	0.115
Triglycerides, mmol/L	1.57±0.94	1.80±1.51	1.80±1.35	1.47±0.61	0.24	0.971	0.847
apolipoprotein A1, g/L	1.13±0.14	1.08±0.17	1.05±0.18	1.06±0.20	7.09	0.069	0.116
apolipoprotein B, g/L	0.83±0.21	0.90±0.22	0.87±0.20	0.84±0.24	3.83	0.280	0.144
Apolipoprotein A1/B	1.48±0.36	1.30±0.35	1.31±0.40	1.40±0.43	5.81	0.121	0.027

*P_1_*, vascular stenosis<10% compared with vascular stenosis 10~50% group

CAD patients were subdivided into different subgroups based on diabetes, New York Heart Association (NYHA) grade, history of myocardial infarction (MI), and number and site of involved vessels, and these subgroups were further analyzed. As shown in Table 3, the PON1 activity and HDL-C levels were significantly lower in the CAD(+)/DM(+), NYHA grade III-IV, and multiple coronary stenosis subgroups. The PON1 activity and HDL-C levels were not significantly different across the different coronary vascular branch subgroups.

**Table 3:** PON1 activity and HDL levels in the CAD subgroup

Categorical variables	Count (100%)	PON1 activity (U/L)	*P* Value	HDL levels (mmol/L)	*P* Value
CAD(+)/DM(+/−)			0.007		0.009
DM+	41	299±96		0.97±0.32	
DM-	82	355±120		1.10±0.31	
CAD(−)/DM(+/−)			0.182		0.382
DM+	10	352±184		1.07±0.18	
DM-	53	385±122		1.16±0.28	
NYHA grade			0.029		0.000
I-II	77	368±120		1.20±0.34	
III-IV	46	318±109		0.98±0.27	
AMI			0.435		0.401
Yes	49	323±96		1.03±0.31	
No	74	345±127		1.08±0.32	
Number of involved vessels			0.005		0.032
single	58	371±123		1.12±0.32	
multi	65	306±100		1.01±0.31	
LAD			0.090		0.516
Yes	102	328±113		1.05±0.30	
No	21	374±120		1.11±0.37	
LCX			0.268		0.451
Yes	53	325±118		1.05±0.37	
No	70	345±114		1.07±0.29	
RCA			0.303		0.228
Yes	77	324±105		1.04±0.33	
No	46	349±125		1.09±0.30	

CAD, Coronary artery disease; DM, Diabetes mellitus; NYHA grade, New York Heart Association classification grading of cardiac function; AMI, acute myocardial infarction; LAD, left anterior descending branch coronary artery; LCX, left circumflex coronary artery; RCA, right coronary artery.

### Factors that affect PON1 activity

Many other factors that affect PON1 activity were assessed, such as age, gender, smoking, alcohol consumption, hypertension, diabetes, serum creatinine, lipid drugs, and lipid levels (Table 4). Age, renal dysfunction and hyperlipidemia are the risk factors of CAD. The standards of these indicators are the most widely used in clinical practice, rather than detailed numbers. So we converted these continuous variables to categorical variables for easy to comparison. Blood lipids were converted and regrouped based on Guidelines on Prevention and Treatment of Blood Lipid Abnormality in Chinese Adults. Age was regrouped based on the incidence of coronary heart disease, whose age of onset is over 45 in male and over 55 in female. Among these factors, age, alcohol consumption, hypertension, and lipid drugs did not affect serum PON1 activity. The PON1 activity level was influenced by gender, smoking, diabetes, and HDL-C levels. The male group had a lower PON1 activity level compared with the female group. Similarly, the smokers had a lower PON1 activity level compared with the non-smokers. Of the various types of lipids, only the HDL-C cholesterol level influenced PON1 activity. The serum PON1 activity was higher in the HDL-C >1 mmol/L group compared with the HDL-C <1 mmol/L group (P<0.05).

**Table 4:** Other factors that affect PON1 activity in addition to CAD

Categorical Variables		Cases (n=186)	PON1 activity, U/L	*P* Value
Age	M>45, F>55	174	347±121	0.169
	M≤45, F≤55	12	406±140	
Gender	male	105	327±112	0.003
	Female	81	383±131	
Smokers	Yes	53	312±106	0.015
	No	133	365±127	
Alcohol consumers	Yes	29	366±115	0.301
	No	157	348±125	
Hypertenion	Yes	117	343±117	0.364
	No	69	364±133	
Diabetes	Yes	51	309±118	0.001
	No	135	367±122	
Lipid drugs	Yes	155	351±121	0.731
	No	31	352±137	
Serum creatinine	<97 umol/L	118	350±121	0.821
	>97 umol/L	68	353±127	
Total cholesterol	<5.2 mmol/L	149	355±125	0.571
	>5.2 mmol/L	37	337±116	
LDL cholesterol	<2.6 mmol/L	96	340±118	0.287
	>2.6 mmol/L	90	362±129	
HDL cholesterol	>1 mmol/L	104	367±122	0.037
	<1 mmol/L	82	331±123	
Triglycerides	<1.7 mmol/L	121	357±124	0.571
	>1.7 mmol/L	65	340±121	

Although the serum creatinine level and myoglobin were significantly higher in the CAD patients compared with the non-CAD patients (Table 1), no correlation was identified between PON1 and creatinine. However, renal function influenced age and HDL-C levels. As shown in Figure 1, creatinine was positive correlated with age and negatively correlated with HDL-C or aopA1. In all, creatinine had different effect on the PON1 activity and HDL-C level, though there was a positive correlation between them. Creatinine affected HDL-C, but not PON1 activity. PON1 activity was not affected by renal function.

### PON1 activity and diabetes mellitus

Univariate regression analysis indicated that the serum PON1 activity was significantly lower in the DM patients compared with the NDM (Table 4). In subgroup studies, there was a significant reduction in PON1 activity along with a decrease in HDL-C among the CAD(+)/DM(+) patients compared with the CAD(+)/DM(−) controls (Table 3). We also analysis PON1 activity and HDL-C in the non-CAD subgroups and found no value between the CAD(−)/DM(+) and CAD(−)/DM(−) patients (Table 3). Additionally, a significantly negative correlation was identified between blood glucose and HDL-C, Apo A1, and Apo A1/B (Figure 1).

### ROC curve analysis for PON1 activity

**Figure 2:**
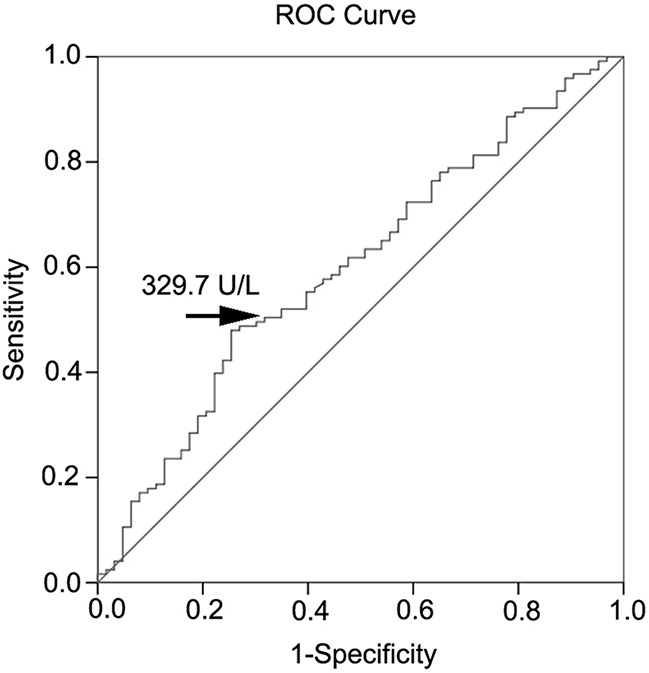
ROC curve analysis of serum PON1 activity

The cut-off levels of PON1 activity used to differentiate between the patients with CAD and non-CAD as determined by the ROC curve analysis are shown in Figure 2 and Table 5. The area under the ROC curve for PON1 activity used to predict CAD was 0.60 (p<0.05). Based on the ROC curve analysis, PON1 activity levels of equal to or lower than 330U/L had remarkable sensitivity and specificity in the prediction of CAD (sensitivity = 52%, specificity = 65%).

**Table 5:** Receiver operating characteristic (ROC) curve for various cutoff levels of PON1 in the differentiation of patients with CAD from non-CAD

PON1 activity (U/L)	Sensitivity (95%CI)	Specificity (95%CI)	DOC
309	0.49 (0.44-0.53)	0.73 (0.69-0.77)	0.58
311	0.50 (0.45-0.54)	0.70 (0.65-0.74)	0.58
330	0.52 (0.48-0.56)	0.65 (0.61-0.70)	0.59(cutoff)
348	0.55 (0.50-0.59)	0.60 (0.56-0.65)	0.60
366	0.62 (0.57-0.66)	0.52 (0.48-0.57)	0.61

* AUC: 0.600 (95% CI: 0.515 – 0.686), *P* =0.026

PON1, serum paraoxonase1; DOC, Distance on curve equaling square root of (1-Sen) ^2^+ (1-Spe) ^2^; AUC, Area under curve

We divided the study population into two groups according to the cut-off value of PON1 activity levels and analyzed the incidence of CAD, risk factors, degree and number of coronary vascular stenosis (Table 6). There were more males, smokers, old agers (M>45, F>55), and increased incidences of CAD (74% vs 59%, P<0.05) and DM (35% vs 21%, P<0.05) in the PON1 activity reduction group. Consistent with our previously described results, the degree and number of coronary vascular stenosis were different between the below and above cut-off point groups. The HDL-C and apopA1 levels were significantly lower in the low PON1 groups; however, there was no significant difference in the other types of lipid levels between the two groups.

**Table 6:** Risk factors predicted by the cut-off value of PON1 activity in the study population

Categorical Variables	PON1 activity (U/L)		
	≥330 (n=100)	<330 (n=86)	*P* value
Age			
M>45, F>55	90 (90%)	84 (98%)	0.034
M≤45, F≤55	10 (10%)	2 (2%)	
Gender			0.012
male	48 (48%)	57 (66%)	
female	52 (52%)	29 (34%)	
Smokers			0.034
Yes	22 (22%)	31 (36%)	
No	78 (78%)	55 (64%)	
Hypertenion			0.783
Yes	62 (62%)	55 (64%)	
No	38 (38%)	31 (36%)	
Diabetes			0.034
Yes	21 (21%)	30 (35%)	
No	79 (79%)	56 (65%)	
CAD			0.027
Yes	59 (59%)	64 (74%)	
No	41 (41%)	22 (26%)	
Degree of Vascular Stenosis			0.017
<10%	24 (24%)	6 (7%)	
10~50%	17 (17%)	16 (19%)	
50~75%	15 (15%)	18 (21%)	
>75%	44 (44%)	46 (53%)	
Number of Involved Vessels			0.027
Without obvious stenosis	41 (41%)	22 (26%)	
Single-vessel>50%	32 (32%)	26 (30%)	
multi-vessel>50%	27 (27%)	38 (44%)	
Total cholesterol, mmol/L	4.34±1.05	4.09±1.07	0.105
LDL cholesterol, mmol/L	2.68±0.87	2.59±0.91	0.462
HDL cholesterol, mmol/L	1.13±0.30	1.04±0.30	0.026
Triglycerides, mmol/L	1.61±0.87	1.60±1.18	0.593
apolipoprotein A1, g/L	1.11±0.19	1.04±0.18	0.021
apolipoprotein B, g/L	0.87±0.23	0.83±0.22	0.272
Apolipoprotein A1/B	1.39±0.42	1.36±0.38	0.860

### Predictive value of decreased PON1 activity concentrations for CAD

We calculated the odds ratio (OR) and 95% confidence interval (95% CI) using univariate and multiple logistic regression analysis to evaluate predictors of CAD for the entire study population (Table 7). PON1 activity (OR 0.495, 95%CI 0.264-0.926, P = 0.028), LDL-C (OR 2.075, 95%CI 1.118-3.853, P = 0.021), and HDL-C (OR 0.508, 95%CI 0.270-0.956, P = 0.036) were predictive in the univariate analysis for CAD. However, only PON1 activity (OR 0.519, 95%CI 0.270-0.998, P = 0.049) was predictive in the multiple analysis for CAD. Age and gender were also predictive in the univariate analysis for CAD, but not on multiple analysis.

**Table 7:** Univariate and multivariate logistic regression model for prediction of coronary artery disease

Variable	Univariate analysis OR (95% CI)	P	Multivariate analysis OR (95% CI)	P
Paraoxonase1 activity, U/L	0.495(0.264-0.926)	0.028	0.519(0.270-0.998)	0.049
Total cholesterol, mmol/L	1.435(0.684-3.009)	0.340	1.225(0.479-3.136)	0.672
LDL cholesterol, mmol/L	2.075(1.118-3.853)	0.021	2.071(0.925-4.635)	0.077
HDL cholesterol, mmol/L	0.508(0.270-0.956)	0.036	0.624(0.276-1.409)	0.256
Triglycerides, mmol/L	1.109(0.588-2.090)	0.749	1.248(0.618-2.523)	0.536
apolipoprotein A1, g/L	0.605(0.319-1.145)	0.123	0.816(0.356-1.867)	0.629
apolipoprotein B, g/L	1.137(0.577-2.243)	0.710	0.534(0.209-1.361)	0.189
Apolipoprotein A1/B	0.729(0.248-2.147)	0.567	0.649(0.180-2.339)	0.509
Age	2.418(1.058-5.525)	0.036	2.169(0.807-5.830)	0.125
Gender	0.478(0.258-0.886)	0.019	0.914(0.407-2.052)	0.828

## DISCUSSION

PON1, which is associated with HDL-C, hydrolyses the oxidized LDL-C and thereby retards the development of atherosclerosis. Many studies have focused on the association of PON1 and CAD and have demonstrated PON1 activity was decreased in CAD, MI and atherosclerosis [14–16]. Consistent with previous findings, our studies indicated the serum PON1 activity level was significantly lower in the patients with CAD compared with non-CAD. By pooling the collected data on PON1 activity and CAD severity, we compared the PON1 activity with different subgroups according to the numbers and degrees of coronary vascular stenosis. The results suggested that a progressive decrease in serum PON1 activity was present for individuals with the severity of coronary lesions. Significantly decreased PON1 activity was noted for patients with two or three vascular stenosis compared with the patients with a single vascular stenosis. PON1 activity decreased as the coronary vascular stenosis degree increased. There was a significant difference in the PON1 activity level between the stenosis <10% and stenosis 10-50% groups. The results suggested that the low PON1 activity level was also present for patients with coronary artery atherosclerosis. The PON1 activity level in the vascular stenosis 50-75% group was lower than the stenosis >75% group; however, this difference was not significant. The reason may be that the limited and unbalanced study population size increased the risk of type I error.

Recent studies have demonstrated serum PON1 activity was significantly lower in patients of AMI, especially at 6 weeks after AMI [17]. However, our study that investigated a CAD subgroup indicated the opposite results. There was no significant difference in PON1 activity between the AMI and no AMI patients, although, the PON1 activity was slightly lower in the AMI patients. Furthermore, there was no significant difference among the different types of coronary vascular branches. However, the PON1 activity level was lower in the heart failure patients compared with the controls. These data suggested the decreased PON1 activity level was a risk factor for multiple coronary vascular lesions and heart failure in CAD.

Diabetes mellitus is a significant and independent predictor of CAD. Studies have demonstrated the PON1 activity was reduced in DM compared with controls [18]. Consistent with previous findings, we demonstrated the serum PON1 activity was significantly lower in the DM patients compared with the non-DM patients. In the subgroup analysis, the CAD(+)/DM(+) patients had significantly lower levels of PON1 activity and HDL-C compared with the CAD(+)/DM(−) patients. High blood glucose affected the serum PON1 activity, which may be related to the decrease in the HDL-C level because the HDL-C level was significantly lower in the CAD(+)/DM(+) subgroup and was negatively correlated with blood glucose. DM is a condition of oxidative stress and resulted in the reduction of PON1 antioxidant activity and the development of atherosclerosis. Thus, decreased PON1 activity is also as a predictor of DM in CAD subjects.

We also assessed other factors that affect the serum PON1 activity. PON1 activity was not associated with age, hypertension, alcohol consumption, creatinine, blood urea nitrogen (BUN), Total cholesterol (TC), Triglycerides (TG) or LDL-C. The serum PON1 activity was significantly lower in the low HDL-C group compared with the normal HDL-C group and positively correlated with the amount of HDL-C, which suggested the PON1 activity level was associated with the amount of HDL-C. In addition, the PON1 activity level was significantly lower in smokers and males. Thus, smoking and gender affected the serum PON1 activity.

The serum PON1 activity levels can predict the severity of coronary vessel involvement. Defining a cut-off value for PON1 activity could help to predict the severity of angiographic lesion. Thus, we determined the cut-point for serum levels of PON1 activity in patients who presented with CAD using the ROC curve. Based on the ROC curve analysis, the laboratory cut-point used to obtain the best predictive results for differentiating the CAD patients from the non CAD patients was 330 U/L (sensitivity = 0.52 and 1-specificity = 0.35). Consequently, we verified the risk factors of CAD using the cut-off standard and demonstrated that the incidence of CAD, age (M>45, F>55), males, smoking, DM, and the degree and number of coronary vascular stenosis were significantly increased in the low PON1 group (PON1 activity < 330 U/L). A clinically significant relationship between PON1 activity and CAD was observed both in univariate and multiple logistic regression analysis. Thus, a serum PON1 activity level below 330U/L may be used as a criterion to predict CAD severity. In Table 7, we compared PON1 activity and other factors for the predictive value of CAD using univariate and multiple logistic regression analysis. The results showed the value of PON1 prediction was 0.028 in univariate logistic regression model and 0.049 in multiple logistic regression model. Of all blood lipid indexes, LDL-C and HDL-C were predictive for CAD in the univariate analysis, but not in multiple analyses. Of course, there are many other factors affected CAD, which we do not study. But our data suggested PON1 activity was a better predictor of CAD than blood lipid.

According to the results of previous studies, PON1 and the lipid level may be affected by lipid drugs [19]. In our study most patients had a history of lipid drug medication. Thus, we compared the level of blood lipid and PON1 activity according to lipid drug medication and determined the HDL-C and PON1 activity were not significantly different between the two groups. Therefore, lipid drugs mainly affected the LDL-C and TC, but not the PON1 activity and HDL-C. The TC and LDL-C levels were significantly lower in the CAD patients compared with the non-CAD patients (Table 1), which may involve the effect of lipid drugs because the CAD patients had a higher lipid drug medication rate than the non-CAD patients.

This study was limited by the small sample size, which only represented a small fraction of all patients admitted to catheterization laboratories throughout Shanghai during the study period. As numerous comparisons were performed with no adjustment for multiple testing, the increased risk of type I error should be acknowledged. Nevertheless, because patient inclusion was consecutive at our hospital, we trust that the survey depicts standard clinical practice.

## CONCLUSION

To summarize, serum PON1 activity played an important role in CAD with multiple coronary lesions and may represent a biochemical marker for CAD severity prior to performing angiography. PON1 activity was associated with the amount of HDL-C and was also affected by blood glucose and smoking.

## MATERIALS AND METHODS

### Study population

A randomized, double-blind contrast test was conducted. All participants were chosen from consenting subjects undergoing an elective, diagnostic cardiac catheterization procedure for acute coronary syndrome or stable angina. We consecutively recruited 186 patients who were referred to the cardiovascular department of our hospital from August 2013 to February 2014. The participants were asked to answer a questionnaire regarding their medical history and regular medication. The data regarding age, gender, history of smoking or alcohol consumer, history of hypertension or diabetes, history of lipid drug medications were recorded according to the survey. Serum lipid metabolism, blood glucose, renal function, myocardial enzyme spectrum were detected in our hospital laboratory. Blood pressure was measured using a calibrated mercury sphygmomanometer in both arms while sitting after at least 30 min of rest, and the average of 3 recordings was used. The study protocol was approved by the Shanghai Ninth People's Hospital Ethics Committee and Research Board. Written informed consent was obtained from all study participants.

### Laboratory determination

All subjects were investigated in the morning after an overnight fast. A venous cannula was inserted in the brachial vein for blood sampling. The blood samples were drawn into 5-ml evacuated and promoting coagulating tubes for biochemical detection, including serum lipid metabolism, blood glucose, and renal function. The blood samples were drawn into 5-ml anticoagulant tubes with heparin for cardiac troponin I and myoglobin detection. Serum was separated via centrifugation, and biochemical measurements were immediately conducted. The serum TC, LDL-C, HDL-C, TG and apolipoprotein (Apo) were enzymatically measured using the Hitachi 747 chemical analyzer (Hitachi, Tokyo, Japan). The LDL-C particle size was determined using gel electrophoresis (Lipoprint TM System; Quantimetrix Corp., Redondo Beach, CA, USA) according to the manufacturer's instructions. Kidney function, cardiac troponin, creatinine kinase and fasting blood glucose were measured using standard laboratory techniques. Serum creatinine was determined by picric acid method (CREA-2, Simens), and Blood urea nitrogen was determined by urease methods (UN Reagents, Simens). Blood glucose was determined by Glucose Oxidase method (GLUH-3, Simens). Myoglobin I and myoglobin were both measured using Chemiluminescence immunoassay (Access AccuTnI+3, Access Myoglobin, Beckman Coulter, USA). CK-MB was determined by an electrochemiluminescence immunoassay method with an Elecsys 2010 Analyser manufactured by Roche Hitachi, Germany 2004. The lower limits of assay measurement were 0.01ng/mL for CK-MB. All assays were conducted by a laboratory scientist according to the manufacturer's specification.

### Analysis of PON1 activity

The second venous blood samples were collected in 5mL blank glass tubes at the time of catheterization prior to angiography for PON1 activity detection. The serum was then separated by centrifugation at 3000 rpm at 4°C for 15 min. Serum samples for all subjects were collected in serum separator tubes, processed, and stored in aliquots at -80°C within 4 hours of phlebotomy until analysis. The serum PON1 activity level was determined using a modification of a spectrophotometry-based assay as previously described [20]. Briefly, the PON1 activity was measured by adding serum to Tris buffer (100 mmol/l, pH 8.0) that contained 2 mmol/l calcium chloride and 5.5 mmol/l paraoxon (O,O-diethyl-O-p-nitrophenylphosphate (Sigma Chemical Co.)). The generation rate of p-nitrophenol was determined at 405 nm, 25°C, with the use of a continuously recording spectrophotometer (Beckman DU-68). The intra- and inter- assay coefficients of variance for the PON1 activity assay used were both < 3.5%, as determined from 30 replicates performed on 15 different days during the course of the sample analyses.

### Diagnostic coronary angiographic examinations

Diagnostic coronary angiography was performed in participants within 6 hours of the diagnosis of MI, within 24 hours of unstable angina, in participants with chronic stable angina who had at least one positive noninvasive test result, and in participants with atypical chest pain speculated as CAD. Angiographies were performed using Judkins right and left catheters through the femoral artery and a Germany's Siemens Axiom Artis DFC ZEE floor-mounted device. The interpretation of the angiographs was performed by two expert cardiologists who were blinded to the blood test results. The quantification analysis of obstructions after catheter calibration was performed by moving the cursor from the proximal through the distal region of each vessel to determine the length and severity of the obstruction. Quantitative coronary angiography (QCA) was performed in biplane views.

The patients were screened for the presence of normal coronary arteries (one or more coronary stenosis <10% in diameter), coronary artery atherosclerosis (one or more coronary stenosis 10 - 50% in diameter), and CAD (one or more coronary stenosis > 50% in diameter) in any three coronary arteries (RCA, LAD, LCX), according to the International Statistical Classification of Diseases and Related Health Problems criteria (10th Revision) and the American Heart Association classification for cardiovascular diseases.

### Statistical analysis

The frequencies of categorical variables are represented as counts (percentages), and the continuous variables are represented as either the mean ± standard deviation or the median with interquartile range. Variables were assessed for normality by Shapiro-Wilk test and Homogeneity of variance test. The baseline characteristics indifferent groups with CAD were compared via Student's *t*-tests (serum lipid metabolism, blood glucose, and renal function) or nonparametric tests (cardiac troponin and myoglobin) for continuous variables and χ^2^ tests for categorical variables (age, gender, smoking, alcohol consumers, the incidence rate of diseases and drugs). Serum PON1 activity levels were non-normal distributions and were analyzed by nonparametric tests and the logistic regression model. Univariate and multivariate analyses was used in evaluating the role of PON1 activity in CAD compared with other CAD risk factors. The Spearman correlation was performed to determine the relationship between the serum paraoxonase1 activity levels and other biochemical parameters. According to the size of the area under the ROC curve (AUC), receiver operating characteristic (ROC) analyses were used to summarize the diagnostic power of PON1 for discrimination between CAD and non-CAD patients. Optimal cut-offs were derived from the ROC curves by maximizing the sum of sensitivity and specificity. Univariate and multiple logistic regression analysis with CAD as a dependent variable were performed to identify the risk factors. Two sided p values less than 0.05 were considered statistically significant. Statistical tests were performed using SPSS (Statistical Package for the Social Sciences) version 17 software (SPSS Inc., Chicago, Illinois, USA).
